# Overactive Bladder Syndrome and the Potential Role of Prostaglandins and Phosphodiesterases: An Introduction

**DOI:** 10.5812/numonthly.14087

**Published:** 2013-09-10

**Authors:** Mohammad Sajjad Rahnama'i, Gommert A. Van Koeveringe, Philip E. Van Kerrebroeck

**Affiliations:** 1 Maastricht University Medical Centre (MUMC+), Maastricht, The Netherlands

**Keywords:** Urinary Bladder, Prostaglandins, Phosphodiesetrase Type 5, Urinary Bladder, Overactive

## Abstract

In this paper, a general introduction is given, presenting the overactive bladder syndrome (OAB) and its impact on the quality of life and economical burden in patients affected. Moreover, the anatomy, physiology and histology of the lower urinary tract are discussed, followed by a brief overview on the possible role of prostaglandin (PG) and phosphodiesterase type 5 (PDE5) in the urinary bladder. The current literature on the role and distribution of PGE_2_ and its receptors in the urinary bladder is discussed. In both animal models and in human studies, high levels of signaling molecules such as PG and cGMP have been implicated, in decreased functional bladder capacity and micturition volume, as well as in increased voiding contraction amplitude. As a consequence, inhibition of prostanoid production, the use of prostanoid receptor antagonists, or PDE inhibitors might be a rational way to treat patients with detrusor overactivity. Similarly, prostanoid receptor agonists, or agents that stimulate their production, might have a function in treating bladder underactivity.

## 1. Introduction

The overactive bladder syndrome (OAB) is defined by the International Continence Society (ICS) as urinary urgency that is accompanied by urinary frequency and nocturia, with or without urgency urinary incontinence (1, 2). OAB has greater impact on people’s quality of life than diabetes (3-6) and an economic burden and cost comparable to rheumatoid arthritis and asthma (7). Therefore, OAB deserves more research resources and research efforts. Those, affected by the symptoms of OAB tend to curtail their participation in social activities e.g. isolate themselves and are predisposed to depression (8). Furthermore, many patients are often too embarrassed to seek medical treatment which contributes to an underestimation of the prevalence and difficulties in understanding the social burden of the disease (3, 9). It is estimated that about 60% of all patients seeking help, experience some symptoms of bladder dysfunction (10). The symptoms encompassing OAB still present a therapeutic challenge. An unmet medical need clearly exists for an effective and well tolerated pharmacological therapy. The current treatment mainly consists of anticholinergic drugs, which have a slightly better effect than placebo, but poor patient compliance, due to the side effects and the lack of sufficient efficacy (11).

Hence, OAB is a major problem affecting a large number of individuals. The underlying causes are not known and the precise mode of action of pharmacological treatments remains unclear. Therefore, new insights into the problem and new therapeutic modalities are urgently needed.

OAB affects nearly 100 million people in the Western world (33 million in the US and 66 million in the European Union) (12, 13) and has severe effects on quality of life and ability to work. OAB is reported to have an incidence of, up to 17% in the Western population 12 and an overall prevalence of 16.6 % in Europe (13). This number is significantly higher in the older population were up to 40% of the individuals over the age of 70 is reported to be affected (13). A recent study has estimated the prevalence of OAB in the United States to range from 26 to 33% in men and from 27 to 46% in women (14).

The total economic cost of OAB is high. In 2002 the costs in the US were approximately $12.7 billion which increased to €22 billion/year in 2005. Approximately 25% of this expenditure, is spent on treatment (drug therapy, clinical consultation, surgery and, incontinence pads). Of those who suffer from OAB, only 28% sought help and only half of those currently receive treatment. Less than 3% of the patients regain long lasting continence. Therefore, the above mentioned costs are likely to be an under-estimation and most probably, the problem is much larger (11-13, 15). As the incidence of OAB increases with age, it will be an increasing problem in aging societies.

The exact economic costs and prevalence of OAB in the Netherlands are unknown. However, it has been calculated that about €200 million are annually spent on protective material such as incontinence pads. In Germany, the direct annual costs have been estimated to be comparable to those of other chronic diseases such as, dementia or diabetes mellitus (16).

A better management of the symptoms of OAB, will improve quality of life, decrease morbidity and disease related costs.

## 2. Overactive Bladder Syndrome 

OAB occurs in both men and women. In some patients, it is accompanied by uncontrolled contractions of the detrusor muscle during bladder filling, called detrusor overactivity (DO). However, patients with OAB do not always present with DO. DO is detected in only about half of patients with OAB by conventional techniques. But, up to 50% of patients presenting with DO on urodynamics, do not complain of clinical symptoms (17, 18). The differences in the relationship between sensation and bladder activity, may be indicative of different clinical states. However, it is more likely that we don’t understand the true nature of the clinical condition yet.

The only currently available tool to link OAB and DO is urodynamics. Nevertheless, DO and OAB share therapeutic options and, partially, common patho-physiological mechanisms (17, 18).

The characteristic symptom of OAB is a strong sudden desire to void, which can not be postponed (urgency). In some patients this sudden desire results in involuntary urine loss, which is called urgency incontinence.

The term frequency refers to an increased number of micturition’s per day (more than 8 times a day) and can occur as a result of reduced functional bladder capacity. Frequency is at least partly caused by patients’ adaptation ('coping mechanism') to avoid leaking urine by maintaining a relatively low urinary volume in the bladder. Patients with OAB usually try to keep their bladder as empty as possible and therefore have more than 8 micturition’s per day in order to maintain continence.

Even a small increase in urgency and/or frequency can have a marked effect on the patient, depending on the timing of these symptoms. Urgency incontinence is reported in around 20% of men and 40% of women with overactive bladder symptoms (15, 19, 20). The diagnosis of OAB is made on the basis of symptoms. However, clinicians could use cystometry to verify uncontrolled bladder contractions during the filling phase and their relation to the OAB symptoms. 

OAB symptoms can be caused by to spinal cord injury or neuronal lesions within the spinal cord or central nervous system which are also seen in neurological diseases such as multiple sclerosis, stroke Parkinson's or Alzheimer's disease. This condition is also known as neurogenic OAB and has a different aetiology as compared to the aforementioned group of patients with OAB, also called idiopathic OAB. While a number of theories have been proposed, the true patho-physiology of OAB remains uncertain and therefore the syndrome of OAB is described as idiopathic. 

## 3. Current Therapies for OAB

Since OAB symptoms have been shown to be associated with detrusor overactivity, it was argued that drugs affecting contractility would alleviate symptoms (21). Activity in the bladder smooth muscle is initiated by muscarinic receptor stimulation. Drugs designed to target these muscarinic receptors are proven to have some effect in decreasing urgency sensation and incontinence. Currently, antimuscarinic drugs are the first-line treatment for OAB. These drugs produce good initial response rates. However, adverse effects and decreasing efficacy cause poor long-term compliance (22). Therefore, it is desirable that alternative treatment methods are developed and made available for patients. At the moment, a lot of research is focused on compounds with potential efficacy for the treatment of OAB some of which are undergoing preclinical and clinical testing. In OAB, there are many potential patho-physiological targets for intervention. This is reflected in the broad variety of mechanisms amongst the agents in development. In general, the new drugs for OAB are being designed to target mucosal (urothelial) signalling, myocyte signalling or aim to modulate the central nervous system.

The focus of therapy has been shifted from the efferent side, trying to reduce muscle activity (e.g. by inhibiting muscarinic receptors) to the afferent side, trying to reduce the afferent outflow by interacting through different peripheral and central signaling molecules. 

This thesis focuses on the local signaling molecules and more specifically on two possible targets for therapy called prostaglandins and phosphodiesterases. 

## 4. Anatomy of the Lower Urinary Tract 

**Figure 1.**
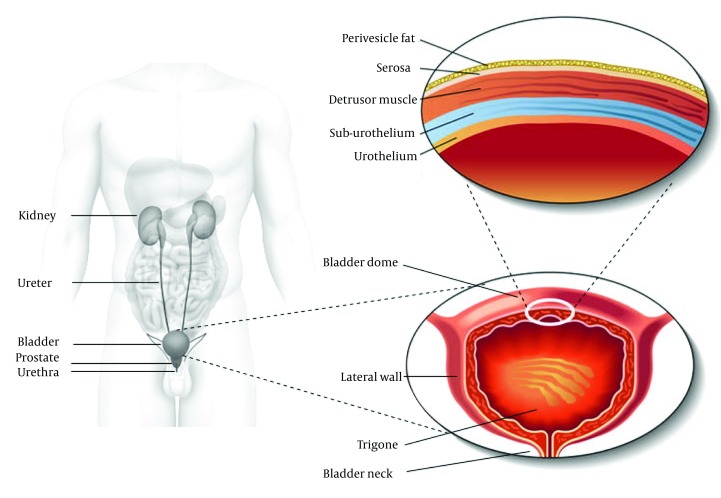
A Schematic View of Bladder Anatomy

The lower urinary tract consists of the distal one third part of the ureters, the bladder and the urethra. These structures are located in the lower pelvis and are supported by muscles and ligaments. The urethra contains both smooth and striated muscles. The bladder can be divided into two main components: the bladder dome, which is located above the ureteral orifices, and the base, consisting of the trigone, urethrovesical junction, lateral wall of the detrusor along with the anterior bladder wall. The bladder is essentially a hollow organ composed of separate layers i.e. the urothelium, the suburothelial layer and the detrusor muscle (Figure 1). The detrusor muscle consists of a complex network of smooth muscle fibers, connective and nervous tissue that is responsible for bladder contractility. The inner lining of the bladder is called the urothelium, which has a barrier function and is also a site of production of many physiologically active molecules. The detrusor muscle is built up by smooth muscle cells and is structurally and functionally different from trigonal and urethral smooth muscle. 

In both men and women, continence is based upon the maintenance of low pressure within the bladder during urine storage and the high tonic tension of the sphincter muscle of the bladder outlet. The latter comprises the smooth muscle of the bladder neck, proximal urethra, and the striated muscle fibers (also referred to as the rhabdosphincter, external urethral sphincter or the striated urethral sphincter) that wrap around the urethra. Furthermore, the pelvic floor muscles are important in the maintenance of urinary continence and in preventing the descent of the pelvic organs.

## 5. Histology of the Bladder Wall

**Figure 2.**
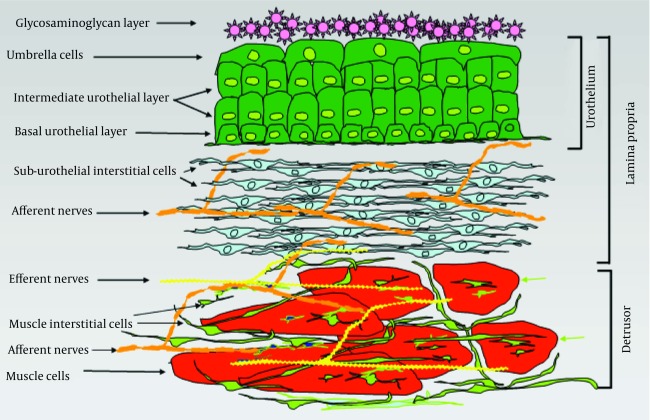
A Schematic View of the Cellular Structure in the Different Bladder Layers

The bladder wall can be divided into three main layers: the urothelium, the suburothelial layer and the muscle layer (Figure 2). The urothelial surface cells are covered by a glycosaminoglycan layer (GAG). The function of the GAG layer remains controversial. Suggested tasks of this layer include an osmotic barrier function and also an antibacterial coating of the urothelium (low adherence of the bacteria) (23).

## 6. Urothelium

The uro-epithelium, better known as urothelium, is the interface between the lumen of the urinary tract and underlying tissues. The urothelium is composed of three layers: a basal cell layer attached to a basement membrane, an intermediate layer, and a superficial or apical layer composed of large hexagonal cells (25-250 μm diameter) called ‘umbrella cells’ (24, 25). The umbrella cells are interconnected by tight junctions. These connections are composed of multiple proteins such as the claudins. The Umbrella cells are covered on their apical surface (nearly 70-80%) by crystalline proteins called uroplakins that assemble into hexagonal plaques (26-28). The urothelium is more than a barrier that separates urine from extracellular fluid. It also has substantial sensory properties (29). Moreover, the lamina propria has been shown to be extensively innervated (30, 31) Receptors for bradykinin, purines (P2X and P2Y) norepinephrine (α and β), acetylocholine (nitrotinic and muscarinic), and neurotrophions have been shown to be present in the urothelium (29, 32-36) as well as a number of transient receptor potential (TRP) channels (TRPV1, 2 and 4) (37, 38). Urothelium is shown to release several substances in response to physical and chemical stimulation. These substances include ATP, nitric oxide (NO), substance P, acetylcholine, PGs and cytokines, (39-43) which suggest the existence of reciprocal communication with neighboring urothelial cells, nerves and interstitial cells. The urothelium has been described as being ‘intelligent’ (44) and is thought to serve as a mechano-sensor, which can control activity in afferent nerves through the production of NO, ATP, acetylcholine and other mediators. Therefore, the urothelium is considered an important part of the bladder wall and a possible target for therapeutic agents.

## 7. Suburothelium

The area between the urothelium and underlying smooth muscle layers is called the suburothelium. This layer is richly supplied by blood vessels, nerves and fibroblasts including myofibroblasts or interstitial cells (IC), which are embedded in a collagen matrix (30, 45). Suburothelial interstitial cells have been identified in human and animal bladders. 

At present, there is still debate on what precisely defines an interstitial cell in the bladder. Several terms have been used to describe cells in the bladder interstitium including; myofibroblasts, interstitial cells of Cajal, and interstitial cells. The initial description of physiologically active interstitial cells in the human and guinea pig bladder was based on their ability to show a rise in cGMP in response to NO donors (46, 47). Interstitial cells have also been subdivided based on their location in the bladder wall. A rough subdivision is made between interstitial cells lying in the suburothelial layer and those in the muscle layer (46). The muscle layer’s interstitial cells are further subdivided. Different cell markers, predominantly c-kit and vimentin, have been used to identify interstitial cells (45, 48-53) Both c-kit and vimentin positive cells are found in the lamina propria and around the muscle bundles of the inner and outer muscle layers. Neither a c-kit, nor vimentin, nor cGMP labeling seem to be ideal for identification of interstitial cells. A large proportion of interstitial cells do not express c-kit (45). Vimentin identifies several interstitial cell types. However, other cell types such as non-physiologically active fibroblasts can express vimentin (45).

Furthermore, cGMP staining identifies, besides interstitial cells, other cells such as umbrella cells and some nerves (47). True identification of cells such as interstitial cells requires investigations of their ultrastructural properties using transmission electron microscopy. Ultrastructural features include: the presence of abundant intermediate filaments, numerous mitochondria, moderately developed Golgi apparatus, smooth endoplasmatic reticulum, contacts with nerves and the formation of gap junctions with each other and with smooth muscle cells (54). The suburothelial interstitial cells found in the bladder are located in close association with afferent nerves, and it has been suggested that these cells play a role in modulating the activity of the suburothelial sensory nerves (50). 50 For example, isolated c-kit positive cells from the suburothelial layer, can respond to stimuli such as caffeine, muscarinic and purinergic agonists with a rise in intracellular calcium concentration (55, 56). Furthermore, vanilloid receptors have been identified on interstitial cells (57).

## 8. Muscle Layer

In many species such as the guinea pig, the bladder muscle is divided in an inner and an outer muscle layer which is surrounded by a thin layer of interstitial cells called the muscle coat. In the guinea pig, three types of muscle interstitial cells have been described based on their location: 1. cells in the outer muscle coat (muscle coat interstitial cells) 2. cells on the surface of the muscle bundles (surface muscle interstitial cells) and 3. cells within the muscle bundles (intra muscle interstitial cells (35, 47, 58). A subpopulation of cells may be identified based on their expression of choline acetyl transferase (59). The muscle interstitial cells are similar to the interstitial cells of Cajal in the gut that mediate peristaltic contractions. Therefore, a pacing role has been suggested for these cells in the bladder wall (60, 61). Interstitial cells maintain close contact with intramural nerves, which might indicate that these cells may be under influence of nerves.

## 9. Bladder Physiology 

As urine is produced in the kidneys, it enters the bladder via the ureters at a rate of 0.5–5 mL/min. The pressure within the bladder (intravesical pressure) remains relatively constant throughout the filling process. During this filling phase the pressure in the urethra must exceed that in the bladder to maintain continence. The ability to maintain a constant low pressure in the bladder during filling is possible as a result of the elastic and visco-elastic properties of the bladder wall, together with activation of neuronal mechanisms that inhibit the detrusor muscle from contracting (62).

The desire to void usually starts when the bladder has reached about half its physiological capacity, after which the desire to void is suppressed by inhibitory input mainly from the cerebral cortex, until an appropriate time and location for micturition has been reached. At the initiation of the micturition process, urethral pressure decreases due to a voluntary relaxation of the urethral sphincter. Moreover, the pelvic floor muscles relax and the bladder neck forms a funnel. Next, increased parasympathetic stimulation causes detrusor muscle contraction and an increased intravesical pressure leads to the voluntary initiation of voiding. After termination of the urine flow, the pelvic floor contracts to elevate and close the bladder neck, urethral pressure increases and pressure within the bladder falls. Any urine within the proximal urethra is forced back into the bladder and bladder filling starts again. The urinary bladder has two control mechanisms, central and peripheral.

## 10. Central Nervous System Control

As the bladder fills during the urine storage phase, the detrusor muscle remains relatively quiescent and the urethral outlet is kept in a contracted state. During the micturition phase, the detrusor muscle contracts and the urethral outlet relaxes. This involves a complex pattern of efferent (motor) and afferent (sensory) signaling in the autonomic and somatic nervous system. The nerves involved are part of a reflex pathway, with an incorporated conscious control component (63). The cerebral cortex, brain stem and spinal cord (S2–S4 segments) are the main structures involved in the regulation of lower urinary tract function. The micturition cycle is initiated in the brain stem, in a region called pontine micturition center. This area is in turn controlled by impulses from the cerebral cortex, which has an inhibitory effect on the detrusor muscle during the filling phase of the bladder.

**Figure 3 .**
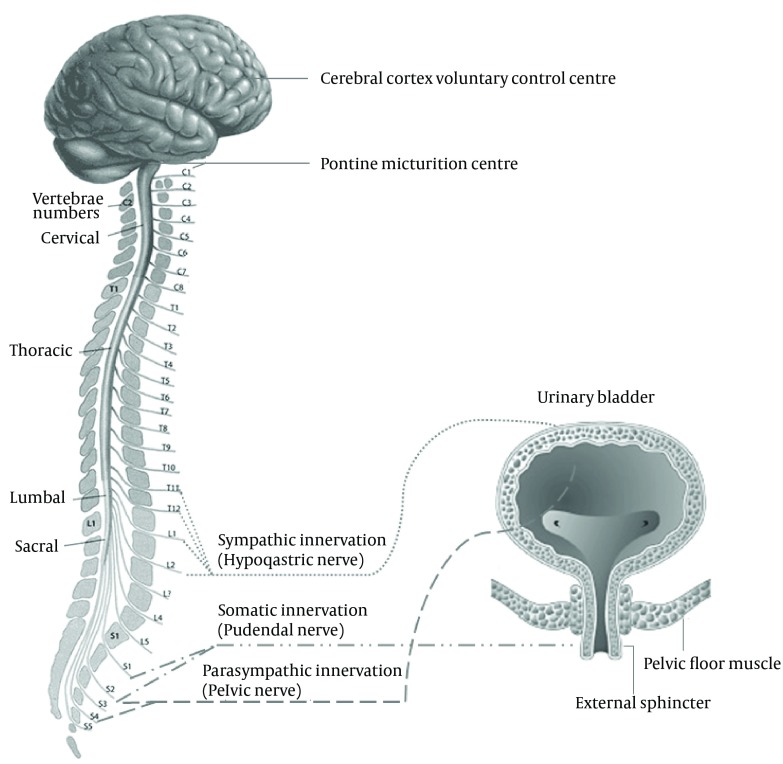
A Schematic View of Bladder Neuronal Control

The lower urinary tract is innervated by both the autonomic (parasympathetic and sympathetic) and somatic nervous systems (Figure 3). Autonomic innervation consists of parasympathetic (pelvic) neurons derived from the S2–S4 segments of the spinal cord and sympathetic (hypogastric) neurons come from the T10–L2 segments. The parasympathetic nervous system mediates the micturition phase and thus the contraction of the detrusor muscle while the sympathetic nervous system contributes to urine storage via relaxation of the detrusor muscle and contraction of the bladder neck and to a lesser extent the urethra (64). The postganglionic neurotransmitter in parasympathetic neurons is acetylcholine, while in postganglionic sympathetic neurons the transmitter is noradrenaline (norepinephrine). Somatic nerves originating from the S2–S4 segments of the spinal cord are responsible for direct innervation of the striated muscle of the urethral sphincter and pelvic floor and have acetylcholine as their neurotransmitter.

## 11. Peripheral Control

The bladder appears to have a peripheral control mechanism located in the bladder wall. This control mechanism is formed by the co-operative interaction of numerous regulatory cell types, of which the interstitial cells and the peripheral neurones are particularly important (65).

In the past decade, there have been a number of advances in our understanding of the detailed structure of the bladder and its more complex functions. One key step forward, was the appreciation that the urothelium released a range of substances when stretched (21). This led to the concept that these chemical agents modify afferent nerve activity and sensation. The urothelium is an integral part of a ‘chemical’ sensory system’ and plays a role in generating and modulating bladder sensation. If the urothelium or sensory nerves become defective, as in for example the OAB syndrome, this would result in increased sensation of urgency. Urothelial cells express various ion channels or receptors that can respond to external substances or mechanical or thermal changes, such as receptors to bradykinin, (15) trkA, and p75 (16); purines (P2X and P2Y); (29) noradrenaline; (33) acetylcholine (nicotinic and muscarinic); (66) protease activated receptors; (67) epithelial Na+ channels (ENaC), (68) the Deg/ENaC family (69) and a number of transient receptor potential (TRP) channels (TRPV1, TRPV2, TRPV4, TRPM8, TRPA1) (70-72). In turn, stimulation of these urothelial sensor molecules can control the release of chemicals, such as ATP, PGs, acetylcholine, and NO, (43) which have excitatory and inhibitory actions on afferent nerves located close to or in the urothelium (72, 73).

A second development was the concept, that phasic activity during the filling phase is likely to be necessary for an adequate bladder sensation.

Although this idea was originally put forward over 100 years ago, its significance as part of a motor-sensory system contributing to the generation of bladder sensation, has only recently been put into a physiological and patho-physiological context (74, 75). The theory proposed, is that altered motor activity, will alter afferent firing and therefore sensation. This idea is embodied in the autonomous bladder hypothesis or theory (76).

As a consequence of this, it is generally accepted that the structure and functions of the bladder are more complex than previously thought. Many laboratories are describing and characterising new cell types, among them, the interstitial cells (47, 48, 77) and neural networks (78). The interstitial cells all play a role in the complex physiological and pathological events. An integral component, generating the non-voiding contractions in the bladder, is the concept that there is a specialized system operating within the bladder wall (75). This system does not involve the post-ganglionic parasympathetic innervation of the detrusor, rather, it is thought to be generated and propagated within a network of specialized cells, the interstitial cells. 

## 12. The Role of Prostaglandins in Bladder Physiology

Arachidonic metabolites, more specifically PGs, are released from the bladder into the general circulation in response to distension (79-81). It was found that PGs in the bladder wall originate from both the urothelial and muscle layers (79, 80). The exact role of this endogenous PG is not known, but it is well documented that exogenous PG alters bladder motor activity in vitro and in vivo and that it can also influence the micturition reflex in humans, (82-84) rats, (85) guinea pigs, (86) rabbits (87) and monkeys (88). The main PGs synthesized in the bladder are PGE_2_ (79-81) and PGI_2_ (89). PGs are locally synthesized in the bladder muscle and mucosa. This synthesis is initiated by stretch of the detrusor muscle, bladder nerve stimulation, bladder mucosa damage and inflammation mediators. The effects of PGs in the bladder have been studied in numerous studies. These studies have shown that bladder infusion with PGE_2_ enhances the micturition reflex and that urine levels of PGE_2_ are increased in patients with OAB (90, 91). Moreover, PG has been suggested to facilitate afferent nerve activity via EP1 receptors during urinary bladder inflammation in rats (92). The level of PGE2 released by bladder strips taken from rats with spinal cord injury was greater than that observed in normal bladders (79). This finding was confirmed in bladders from patients with neurogenic overactive bladder or detrusor overactivity due to spinal cord injury compared to healthy controls (79). Furthermore, the increased number and amplitude of non-voiding bladder contractions resulting from spinal cord injury in rats could be counteracted by the administration of an EP1 receptor antagonist. This antagonist also increased micturition volume (79). More data supporting the involvement of PGE_2_ in detrusor activity came from a study showing that intravesical instillation with PGE_2_ increased frequency of micturition and increased basal intravesical pressure in normal, conscious rats (93).

**Figure 4.**
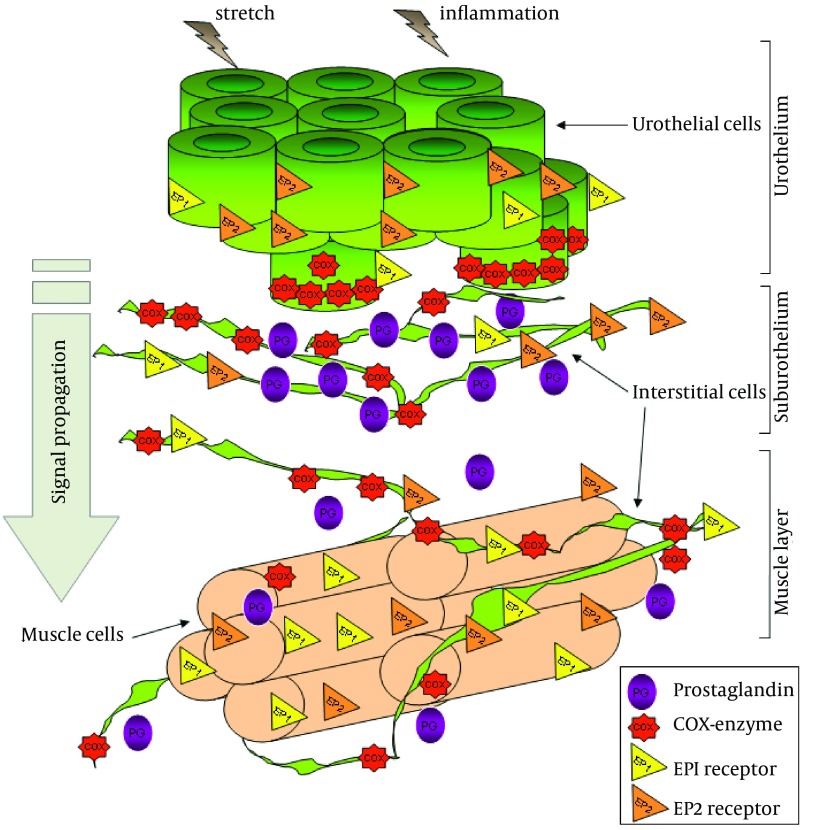
A Schematic View of the Prostaglandin System in the Bladder

Although the role of EP1 receptors on bladder function has been subject of different studies, (92, 93) the cellular localization and the expression of EP receptors in the bladder wall remain unknown. This information would provide interesting data which would help our understanding of how these receptors are involved in the regulation of the micturition reflex. Regarding the actions of PG on the smooth muscle, it was thought, that PG might be co-released with acetylcholine at efferent nerve endings and so, directly contribute to muscle excitation (88). Alternatively, PG might act indirectly on pre-synaptic motor terminals to affect the release of excitatory transmitters (85). It has also been postulated that PG might also inhibit acetylcholine esterase (84) or enhance myogenic bladder activity (94). In regard to PG related changes in the micturition reflex, it was envisaged that they might act directly on the afferent nerves to modulate firing and so, trigger micturition at lower bladder volumes (95). Such an action could arise anywhere in the bladder wall where PG is synthesized and where there are afferent nerves. One specific location where this could occur is the urothelium and suburothelial layers. It is conceivable that they could act upon suburothelial nerve fibers. This would represent a similar situation to that proposed for urothelial derived ATP and its subsequent activation of afferent nerves (52). The idea that PG directly affects afferent firing has yet to be demonstrated. In the guinea pig, it has recently been shown that the enzymes cyclo-oxygenase type 1 (COX-1) and cyclo-oxygenase type 2 (COX-2) are located within specific cell types within the lamina propria. COX-1 is found in the basal layers of the urothelium and associated with the distributed network of lamina propria interstitial cells (96). In contrast, COX-2 is associated with the nuclei of a population of umbrella cells and the nuclei of the suburothelial interstitial cells. Thus, PG signaling in this region of the bladder wall may be complex with multiple sites of production and sites of action (Figure 4).

## 13. Prostaglandins in the Treatment of OAB 

PG is known to be increased in the urine of patients with pathological bladder conditions, including OAB (97). These findings have generated interest in PG as a possible therapeutic target for OAB. There is also interest in the use of inhibitors of the COX enzyme which is an essential enzyme in the conversion of arachidonic acid to PGs. The use of non-selective COX inhibitors improved neurogenic detrusor overactivity in cerebrally infarcted rats (98). In this regard, modulation of PG receptors would be another interesting target possibly through the peripheral afferent systems in the bladder wall. The efficacy of COX inhibitors and PG receptor antagonists in decreasing muscular tone and spontaneous contractile activity of the bladder has been confirmed repeatedly in various animal model studies (99-101). Intravesical instillations with aspirin in partially obstructed rabbit bladders attenuated, albeit not significantly, the contractile response to cholinergic stimulation (102). Moreover, the effects of a nonselective COX inhibitor (dexketoprofen) and a selective COX 2 inhibitor (NS 398) have been studied in both normal and inflamed bladders (99). The results suggest that modulation of the PG production via COX 1, might modulate the threshold for the activation of the micturition reflex in normal rats and that inhibiting COX 2 could prevent the urodynamic changes associated with bladder inflammation (99).

## 14. The Role of NO-cGMP in Bladder Physiology 

Research in Maastricht has shown that interstitial cells responded to exogenous NO with a rise in cGMP (46). Later, these findings were extended, demonstrating that the network of interstitial cells consists of several cell types: cells in the suburothelium (suburothelial interstitial cells; Su-ICs), cells which lie on the surface of the smooth muscle bundles (superficial muscle interstitial cells; SM-ICs) and cells which lie within the muscle bundles (intra-muscular interstitial cells IM-ICs). Examples of these cells are shown in several studies (31, 47, 51-53, 59, 103-106). Most direct evidence that these interstitial cells are responsible for this phasic activity comes from work in the mouse. In the mouse bladder, the only NO responsive cells appear to be the interstitial cells, associated with the outer muscle layer (107). Phasic activity can be generated in these preparations, using muscarinic agonists. Application of NO completely inhibits this muscarinic induced phasic activity. 

From this, it can be concluded that the muscle interstitial cells are involved in the generation of this phasic activity and that a rise in cGMP inhibits this phasic activity. Interstitial cells in the bladder can respond to NO and influence contraction. They are thought to have the characteristics of a network, with pacemaker and conducting elements (108). Furthermore, it is known that the bladder urothelium and suburothelial cells (mucosa) from several different species, including human, releases a number of substances that have an inhibitory effect on smooth muscle contractility and include NO, PGs, and adenine nucleotides. However, there is no clear understanding of the role that these substances play in physiological or patho-physiological control of bladder contractility. In muscle bath studies of several different animal species, surgical removal of the mucosal layer increases the contractile response to several different agonists (73). This indicates that the mucosa releases agents that depress muscle contractility and metabolizes or otherwise inactivates these agonists. Furthermore, the mucosa acts as a barrier to diffusion and penetration of agonists into the muscle or responds to the agonists through the release of substances that reduce the contraction of the underlying muscle.

During the voiding phase, the NO-cGMP signaling pathway is activated in men, allowing efficient voiding through the relaxation of the urethra and prostate (109). Systemic NO augmentation lowers functional bladder outlet resistance very rapidly in men and the NO-cGMP pathway may be a target in the treatment of lower urinary tract symptoms (109).

## 15. Phosphodiesterase Inhibitors in the Treatment of OAB

Phosphodiesterase (PDE) inhibitors prevent the degradation of cyclic guanosine monophosphate (cGMP) and cyclic adenosine monophosphate (cAMP), which are important mediators in maintaining smooth muscle tone. PDE inhibitors were originally designed to treat cardiovascular disease and were later used for the treatment of erectile dysfunction. Recently, they have been shown to be of potential therapeutic use in the treatment of OAB and male LUTS. There are 11 members in the family of PDE-inhibitors characterized and three more are suggested to exist (110-112). Initial evidence regarding the utilisation of PDE inhibitors originates from a study of 19 patients with refractory urgency incontinence (113). Patients were treated with a PDE1 inhibitor, vinpocetine, and 58% of these patients demonstrated an improvement in either clinical or urodynamic parameters. In vitro and in vivo studies are on-going since PDE inhibitors are a logic treatment for OAB. Another recent development has been the finding that PDE type 5 inhibitors (sildenafil, tadalafil and vardenafil) improve urinary symptoms in men with erectile dysfunction and OAB, and this finding has been confirmed in several well-designed randomized controlled trials (114-117). The PDE5-enzyme breaks down cGMP which is a central signal molecules affecting particular cellular functions in the bladder. PDE5-inhibitors, inhibit the PDE5-enzyme, resulting in an increase in intracellular cGMP. Despite the potential benefits of PDE5-inhibitors in OAB, the mechanism of action on the bladder is not known yet. It was demonstrated in the anaesthetised rat, that the PDE5-inhibitor vardenafil, has an inhibitory effect on the non-voiding contractions during the filling phase, suggesting an action on the smooth muscle (118, 119). Furthermore, it has been demonstrated, that PDE5-inhibitors can reduce the force generated in strips of the detrusor muscle (119). In this paper, it was also reported that the PDE5-enzyme was located within the muscle fibers. Using these data, it was argued that the observed muscle relaxation due to PDE5 inhibition was the result of a raised cGMP, resulting in inhibition of detrusor contraction. 

However, there are several published reports, which demonstrate that, after stimulation of the production of cGMP by NO, the muscle cells of human, mice and guinea pig bladders do not respond with a rise in cGMP (46, 47, 107). Nonetheless, a rise in cGMP does occur in cells of a different type, lying in between the muscle cells, the so called, interstitial cells. Based on these observations, it can be argued, that NO doesn’t have a direct relaxing effect on the detrusor muscle, through a rise in cGMP. Instead, it is hypothesized that the aforementioned NO excitable interstitial cells play a role in the regulation of the bladder contractions and that these might be the site of PDE inhibitor action in the bladder.
